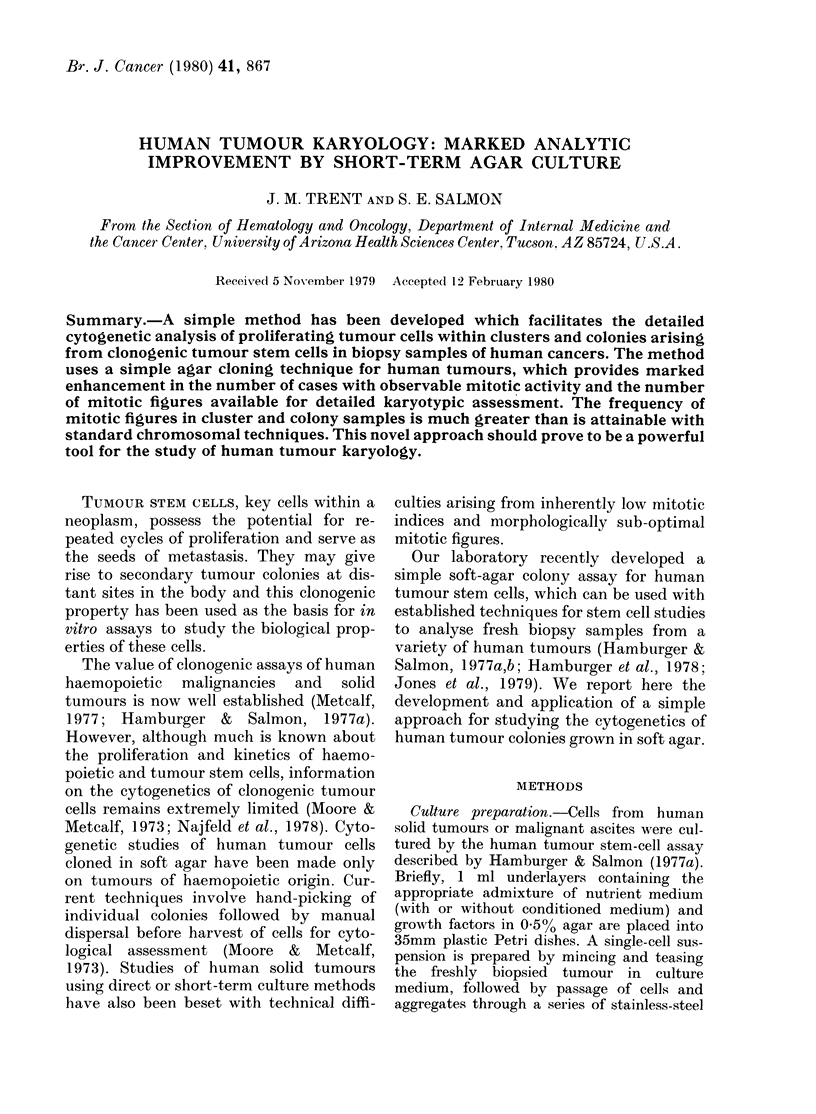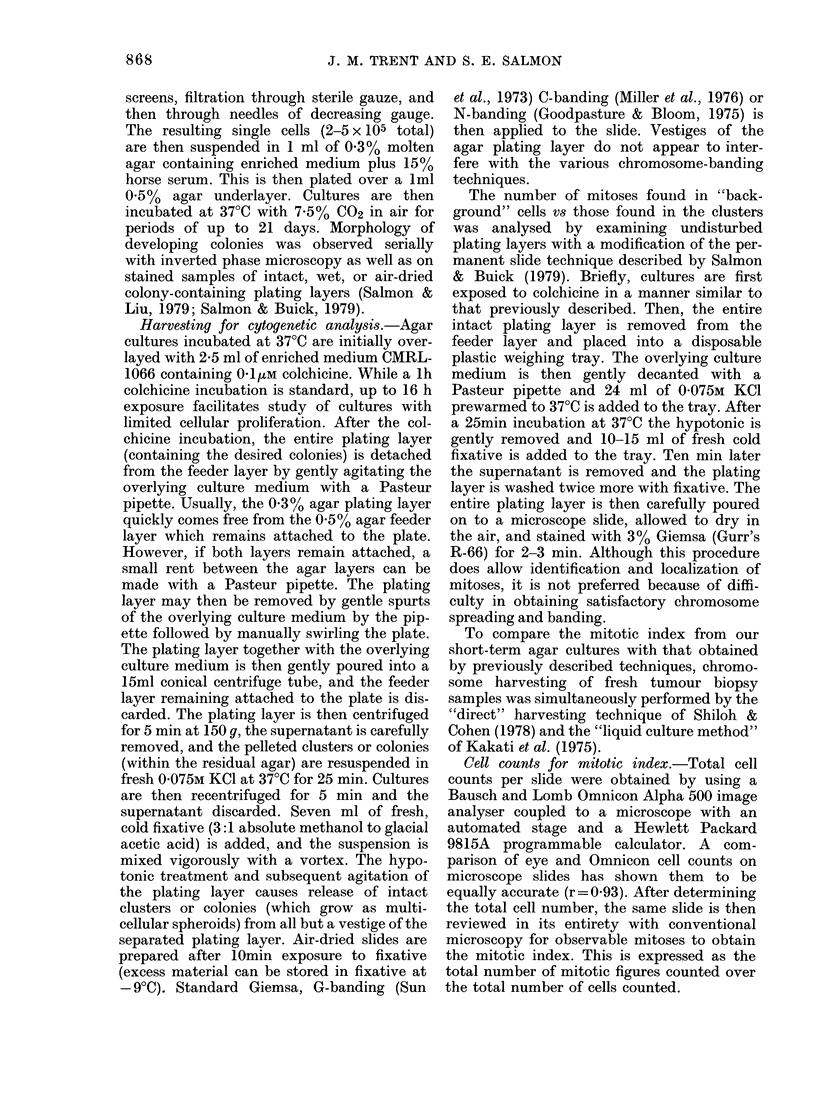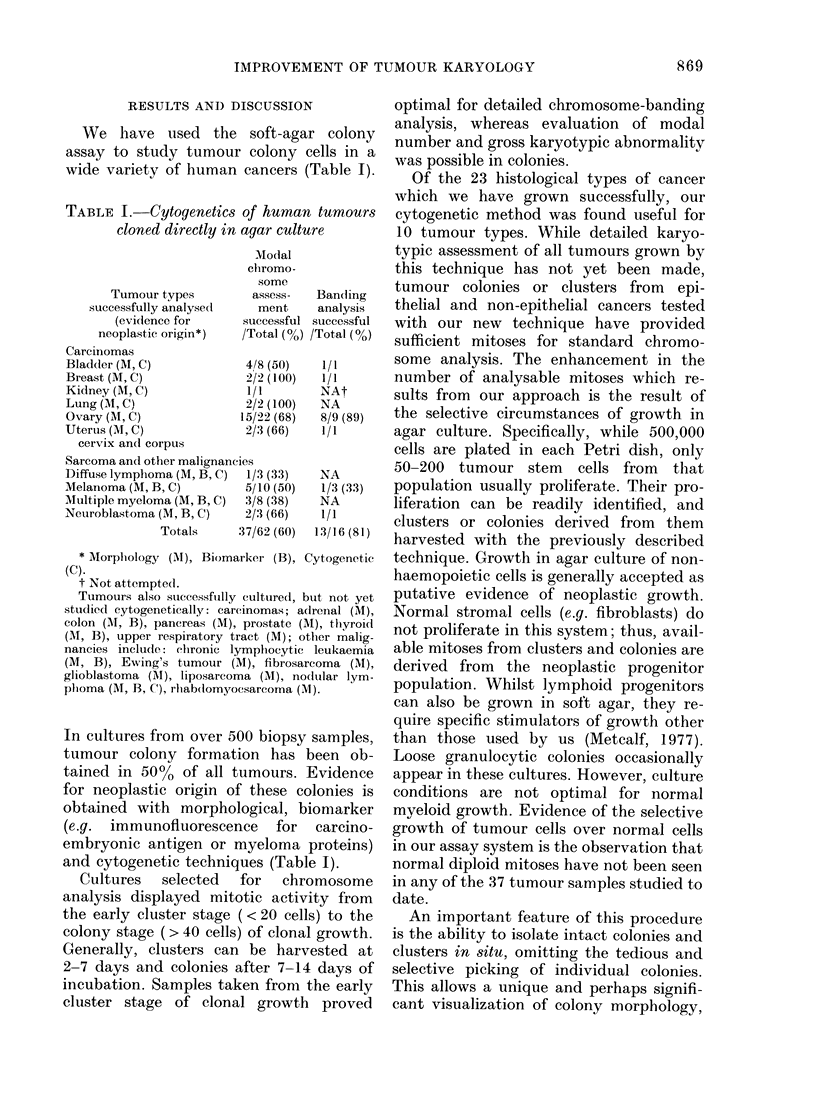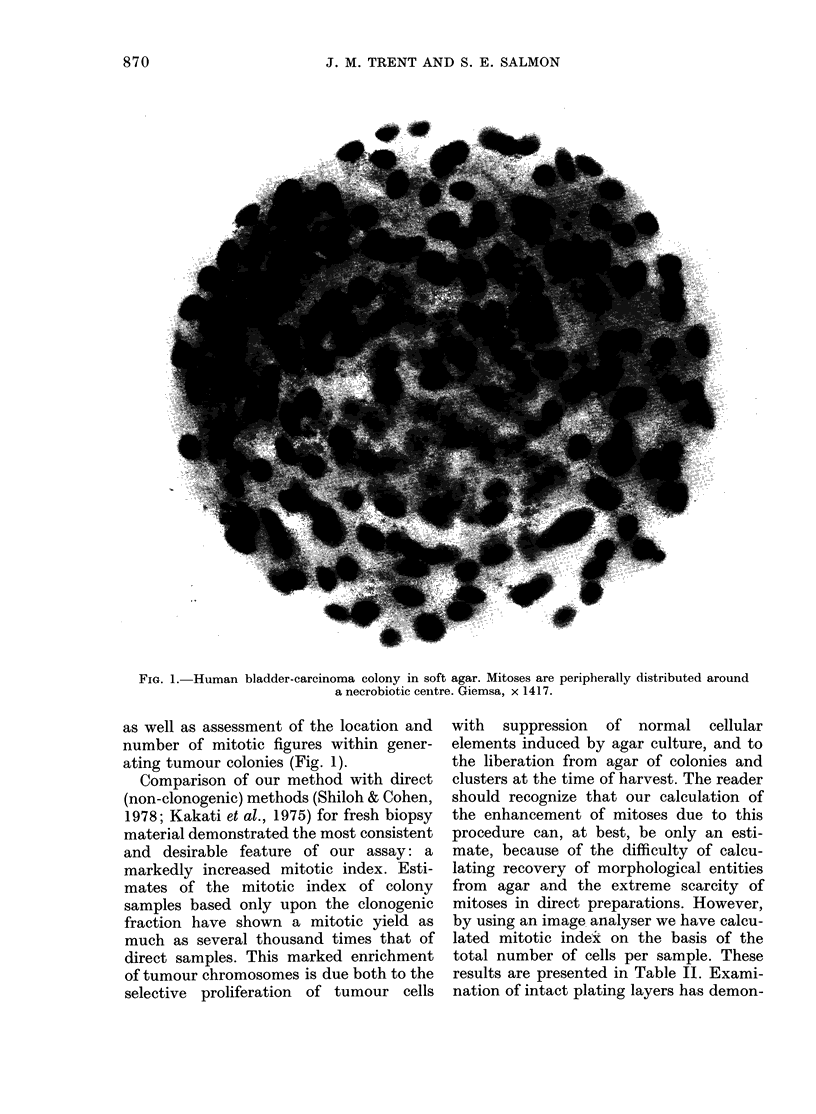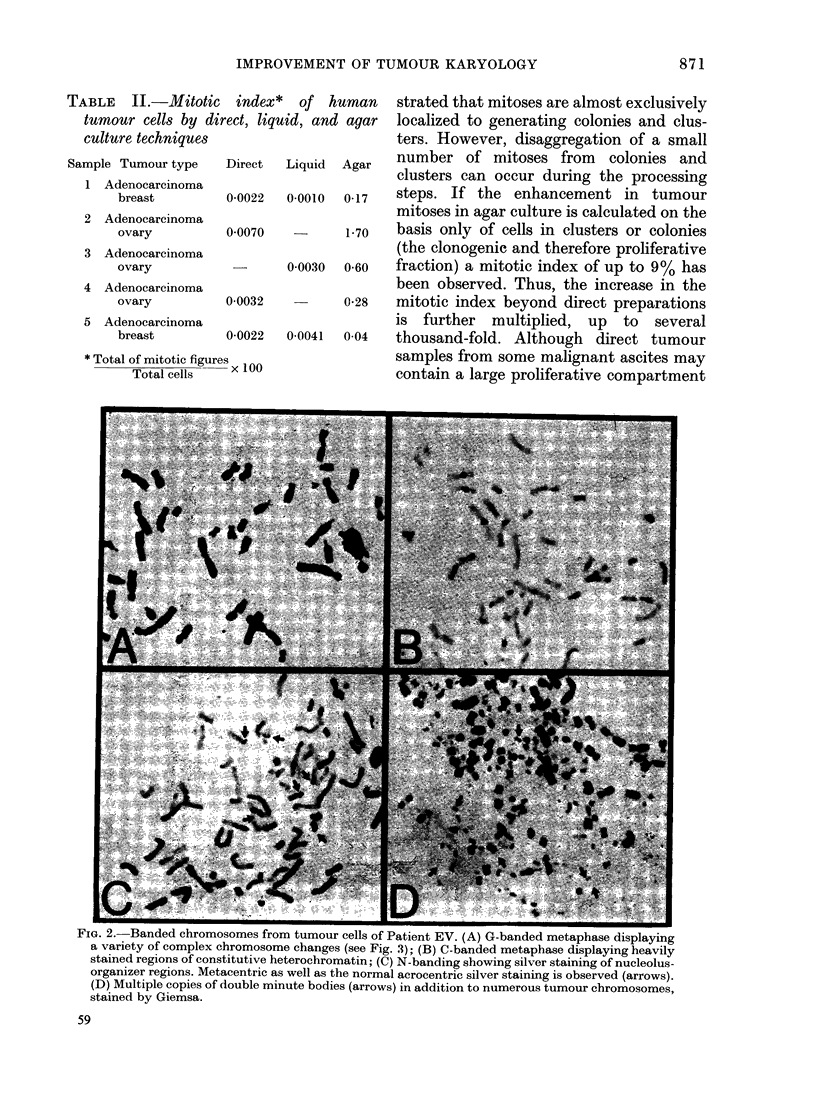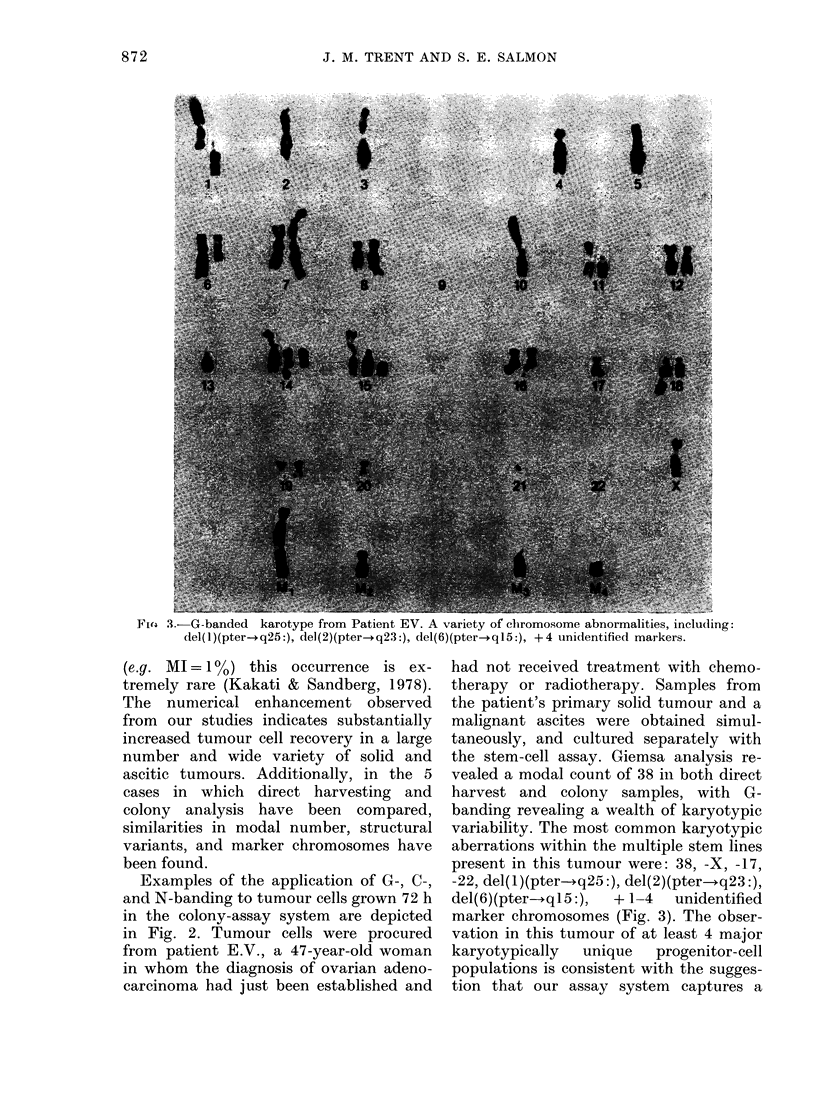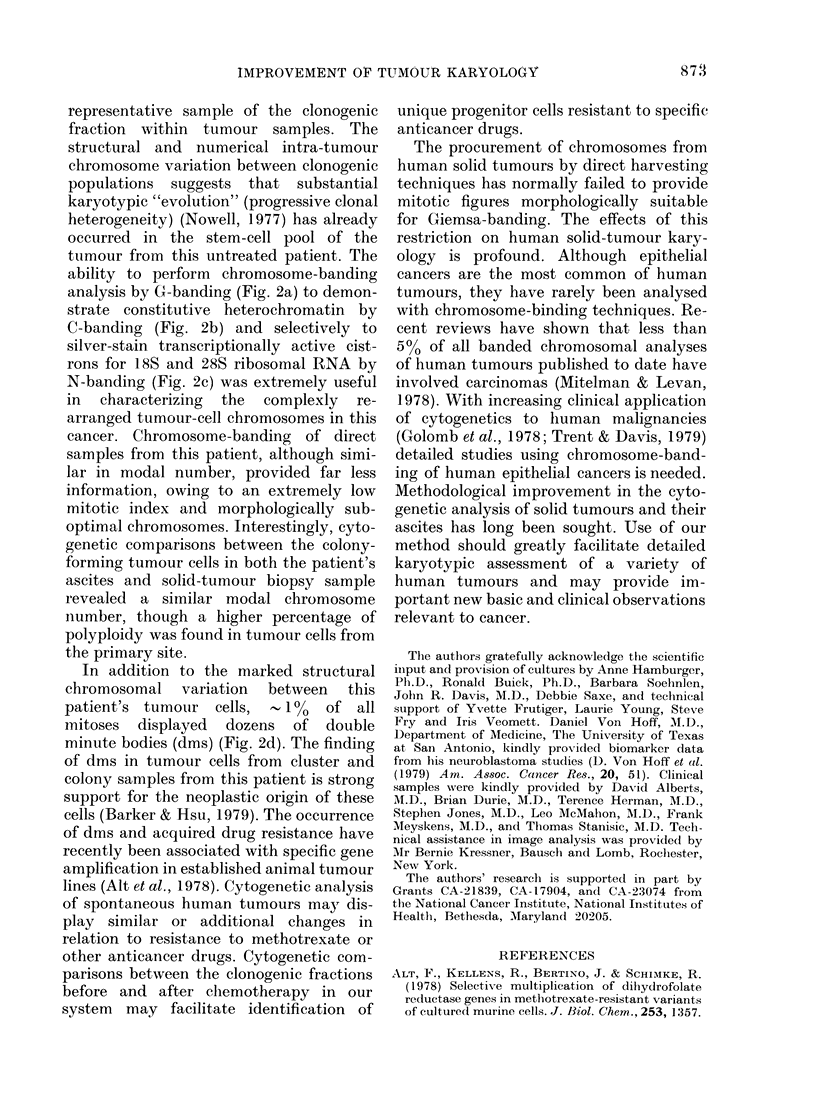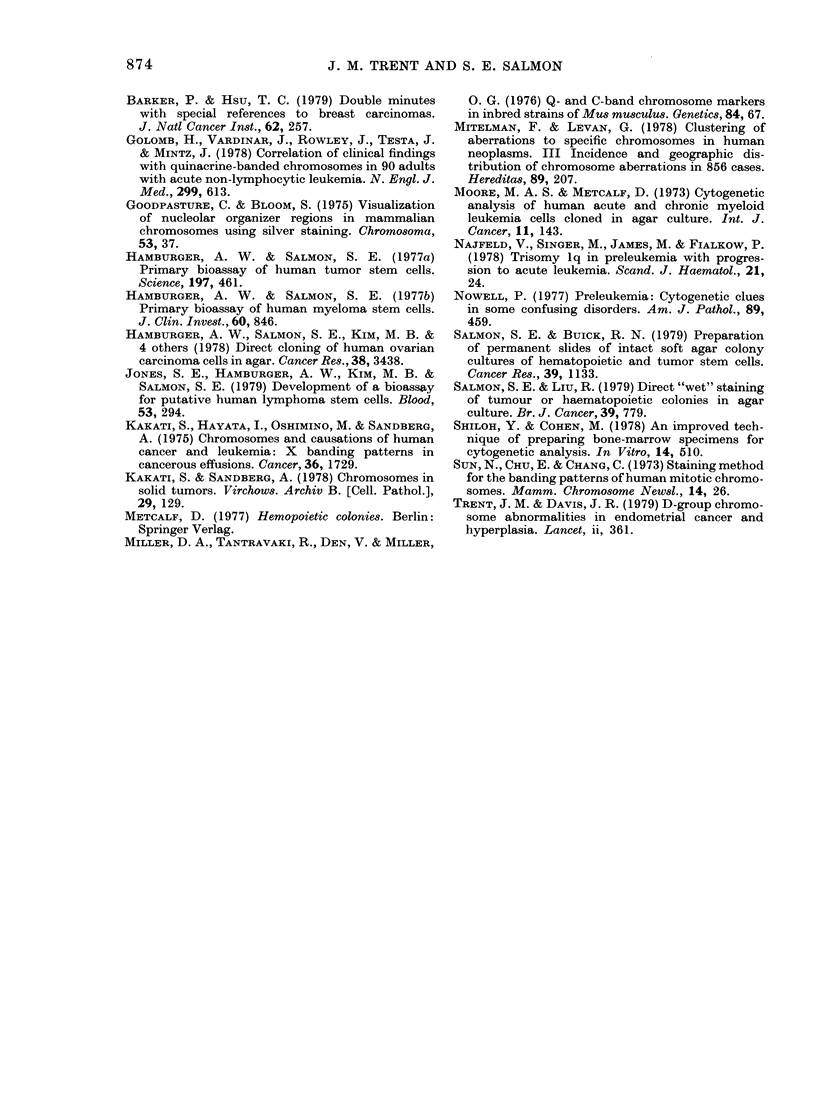# Human tumour karyology: marked analytic improvement by short-term agar culture.

**DOI:** 10.1038/bjc.1980.162

**Published:** 1980-06

**Authors:** J. M. Trent, S. E. Salmon

## Abstract

**Images:**


*Br. J. Cancer* (1980) **41**, 867

### HUMAN TUMOUR KARYOLOGY: MARKED ANALYTIC IMPROVEMENT BY SHORT-TERM AGAR CULTURE

J. M. TRENT AND S. E. SALMON

*From the Section of Hematology and Oncology, Department of Internal Medicine and the Cancer Center, University of Arizona Health Sciences Center, Tucson, AZ* 85724, *U.S.A.*

Received 5 November 1979 Accepted 12 February 1980

**Summary.—A simple method has been developed which facilitates the detailed cytogenetic analysis of proliferating tumour cells within clusters and colonies arising from clonogenic tumour stem cells in biopsy samples of human cancers. The method uses a simple agar cloning technique for human tumours, which provides marked enhancement in the number of cases with observable mitotic activity and the number of mitotic figures available for detailed karyotypic assessment. The frequency of mitotic figures in cluster and colony samples is much greater than is attainable with standard chromosomal techniques. This novel approach should prove to be a powerful tool for the study of human tumour karyology.**

TUMOUR STEM CELLS, key cells within a neoplasm, possess the potential for repeated cycles of proliferation and serve as the seeds of metastasis. They may give rise to secondary tumour colonies at distant sites in the body and this clonogenic property has been used as the basis for *in vitro* assays to study the biological properties of these cells.

The value of clonogenic assays of human haemopoietic malignancies and solid tumours is now well established (Metcalf, 1977; Hamburger & Salmon, 1977*a*). However, although much is known about the proliferation and kinetics of haemopoietic and tumour stem cells, information on the cytogenetics of clonogenic tumour cells remains extremely limited (Moore & Metcalf, 1973; Najfeld *et al.*, 1978). Cytogenetic studies of human tumour cells cloned in soft agar have been made only on tumours of haemopoietic origin. Current techniques involve hand-picking of individual colonies followed by manual dispersal before harvest of cells for cytological assessment (Moore & Metcalf, 1973). Studies of human solid tumours using direct or short-term culture methods have also been beset with technical difficulties arising from inherently low mitotic indices and morphologically sub-optimal mitotic figures.

Our laboratory recently developed a simple soft-agar colony assay for human tumour stem cells, which can be used with established techniques for stem cell studies to analyse fresh biopsy samples from a variety of human tumours (Hamburger & Salmon, 1977*a*,*b*; Hamburger *et al.*, 1978; Jones *et al.*, 1979). We report here the development and application of a simple approach for studying the cytogenetics of human tumour colonies grown in soft agar.

### METHODS

*Culture preparation.*—Cells from human solid tumours or malignant ascites were cultured by the human tumour stem-cell assay described by Hamburger & Salmon (1977*a*). Briefly, 1 ml underlayers containing the appropriate admixture of nutrient medium (with or without conditioned medium) and growth factors in 0·5% agar are placed into 35mm plastic Petri dishes. A single-cell suspension is prepared by mincing and teasing the freshly biopsied tumour in culture medium, followed by passage of cells and aggregates through a series of stainless-steel screens, filtration through sterile gauze, and then through needles of decreasing gauge. The resulting single cells ($2–5 \times 10^5$ total) are then suspended in 1 ml of 0·3% molten agar containing enriched medium plus 15% horse serum. This is then plated over a 1ml 0·5% agar underlayer. Cultures are then incubated at 37°C with 7·5% $CO_2$ in air for periods of up to 21 days. Morphology of developing colonies was observed serially with inverted phase microscopy as well as on stained samples of intact, wet, or air-dried colony-containing plating layers (Salmon & Liu, 1979; Salmon & Buick, 1979).

*Harvesting for cytogenetic analysis.*—Agar cultures incubated at 37°C are initially overlayed with 2·5 ml of enriched medium CMRL-1066 containing 0·1$\mu$M colchicine. While a 1h colchicine incubation is standard, up to 16 h exposure facilitates study of cultures with limited cellular proliferation. After the colchicine incubation, the entire plating layer (containing the desired colonies) is detached from the feeder layer by gently agitating the overlying culture medium with a Pasteur pipette. Usually, the 0·3% agar plating layer quickly comes free from the 0·5% agar feeder layer which remains attached to the plate. However, if both layers remain attached, a small rent between the agar layers can be made with a Pasteur pipette. The plating layer may then be removed by gentle spurts of the overlying culture medium by the pipette followed by manually swirling the plate. The plating layer together with the overlying culture medium is then gently poured into a 15ml conical centrifuge tube, and the feeder layer remaining attached to the plate is discarded. The plating layer is then centrifuged for 5 min at 150 *g*, the supernatant is carefully removed, and the pelleted clusters or colonies (within the residual agar) are resuspended in fresh 0·075M KCl at 37°C for 25 min. Cultures are then recentrifuged for 5 min and the supernatant discarded. Seven ml of fresh, cold fixative (3:1 absolute methanol to glacial acetic acid) is added, and the suspension is mixed vigorously with a vortex. The hypotonic treatment and subsequent agitation of the plating layer causes release of intact clusters or colonies (which grow as multicellular spheroids) from all but a vestige of the separated plating layer. Air-dried slides are prepared after 10min exposure to fixative (excess material can be stored in fixative at −9°C). Standard Giemsa, G-banding (Sun *et al.*, 1973) C-banding (Miller *et al.*, 1976) or N-banding (Goodpasture & Bloom, 1975) is then applied to the slide. Vestiges of the agar plating layer do not appear to interfere with the various chromosome-banding techniques.

The number of mitoses found in "background" cells *vs* those found in the clusters was analysed by examining undisturbed plating layers with a modification of the permanent slide technique described by Salmon & Buick (1979). Briefly, cultures are first exposed to colchicine in a manner similar to that previously described. Then, the entire intact plating layer is removed from the feeder layer and placed into a disposable plastic weighing tray. The overlying culture medium is then gently decanted with a Pasteur pipette and 24 ml of 0·075M KCl prewarmed to 37°C is added to the tray. After a 25min incubation at 37°C the hypotonic is gently removed and 10–15 ml of fresh cold fixative is added to the tray. Ten min later the supernatant is removed and the plating layer is washed twice more with fixative. The entire plating layer is then carefully poured on to a microscope slide, allowed to dry in the air, and stained with 3% Giemsa (Gurr's R-66) for 2–3 min. Although this procedure does allow identification and localization of mitoses, it is not preferred because of difficulty in obtaining satisfactory chromosome spreading and banding.

To compare the mitotic index from our short-term agar cultures with that obtained by previously described techniques, chromosome harvesting of fresh tumour biopsy samples was simultaneously performed by the "direct" harvesting technique of Shiloh & Cohen (1978) and the "liquid culture method" of Kakati *et al.* (1975).

*Cell counts for mitotic index.*—Total cell counts per slide were obtained by using a Bausch and Lomb Omnicon Alpha 500 image analyser coupled to a microscope with an automated stage and a Hewlett Packard 9815A programmable calculator. A comparison of eye and Omnicon cell counts on microscope slides has shown them to be equally accurate ($r = 0{\cdot}93$). After determining the total cell number, the same slide is then reviewed in its entirety with conventional microscopy for observable mitoses to obtain the mitotic index. This is expressed as the total number of mitotic figures counted over the total number of cells counted.

### RESULTS AND DISCUSSION

We have used the soft-agar colony assay to study tumour colony cells in a wide variety of human cancers (Table I).

TABLE I.—*Cytogenetics of human tumours cloned directly in agar culture*

| Tumour types successfully analysed (evidence for neoplastic origin*) | Modal chromosome assessment successful /Total (%) | Banding analysis successful /Total (%) |
|---|---|---|
| Carcinomas | | |
| Bladder (M, C) | 4/8 (50) | 1/1 |
| Breast (M, C) | 2/2 (100) | 1/1 |
| Kidney (M, C) | 1/1 | NA† |
| Lung (M, C) | 2/2 (100) | NA |
| Ovary (M, C) | 15/22 (68) | 8/9 (89) |
| Uterus (M, C) cervix and corpus | 2/3 (66) | 1/1 |
| Sarcoma and other malignancies | | |
| Diffuse lymphoma (M, B, C) | 1/3 (33) | NA |
| Melanoma (M, B, C) | 5/10 (50) | 1/3 (33) |
| Multiple myeloma (M, B, C) | 3/8 (38) | NA |
| Neuroblastoma (M, B, C) | 2/3 (66) | 1/1 |
| Totals | 37/62 (60) | 13/16 (81) |

* Morphology (M), Biomarker (B), Cytogenetic (C).

† Not attempted.

Tumours also successfully cultured, but not yet studied cytogenetically: carcinomas; adrenal (M), colon (M, B), pancreas (M), prostate (M), thyroid (M, B), upper respiratory tract (M); other malignancies include: chronic lymphocytic leukaemia (M, B), Ewing's tumour (M), fibrosarcoma (M), glioblastoma (M), liposarcoma (M), nodular lymphoma (M, B, C), rhabdomyocsarcoma (M).

In cultures from over 500 biopsy samples, tumour colony formation has been obtained in 50% of all tumours. Evidence for neoplastic origin of these colonies is obtained with morphological, biomarker (*e.g.* immunofluorescence for carcinoembryonic antigen or myeloma proteins) and cytogenetic techniques (Table I).

Cultures selected for chromosome analysis displayed mitotic activity from the early cluster stage (< 20 cells) to the colony stage (> 40 cells) of clonal growth. Generally, clusters can be harvested at 2–7 days and colonies after 7–14 days of incubation. Samples taken from the early cluster stage of clonal growth proved optimal for detailed chromosome-banding analysis, whereas evaluation of modal number and gross karyotypic abnormality was possible in colonies.

Of the 23 histological types of cancer which we have grown successfully, our cytogenetic method was found useful for 10 tumour types. While detailed karyotypic assessment of all tumours grown by this technique has not yet been made, tumour colonies or clusters from epithelial and non-epithelial cancers tested with our new technique have provided sufficient mitoses for standard chromosome analysis. The enhancement in the number of analysable mitoses which results from our approach is the result of the selective circumstances of growth in agar culture. Specifically, while 500,000 cells are plated in each Petri dish, only 50–200 tumour stem cells from that population usually proliferate. Their proliferation can be readily identified, and clusters or colonies derived from them harvested with the previously described technique. Growth in agar culture of non-haemopoietic cells is generally accepted as putative evidence of neoplastic growth. Normal stromal cells (*e.g.* fibroblasts) do not proliferate in this system; thus, available mitoses from clusters and colonies are derived from the neoplastic progenitor population. Whilst lymphoid progenitors can also be grown in soft agar, they require specific stimulators of growth other than those used by us (Metcalf, 1977). Loose granulocytic colonies occasionally appear in these cultures. However, culture conditions are not optimal for normal myeloid growth. Evidence of the selective growth of tumour cells over normal cells in our assay system is the observation that normal diploid mitoses have not been seen in any of the 37 tumour samples studied to date.

An important feature of this procedure is the ability to isolate intact colonies and clusters *in situ*, omitting the tedious and selective picking of individual colonies. This allows a unique and perhaps significant visualization of colony morphology,

FIG. 1.—Human bladder-carcinoma colony in soft agar. Mitoses are peripherally distributed around a necrobiotic centre. Giemsa, × 1417.

as well as assessment of the location and number of mitotic figures within generating tumour colonies (Fig. 1).

Comparison of our method with direct (non-clonogenic) methods (Shiloh & Cohen, 1978; Kakati *et al.*, 1975) for fresh biopsy material demonstrated the most consistent and desirable feature of our assay: a markedly increased mitotic index. Estimates of the mitotic index of colony samples based only upon the clonogenic fraction have shown a mitotic yield as much as several thousand times that of direct samples. This marked enrichment of tumour chromosomes is due both to the selective proliferation of tumour cells with suppression of normal cellular elements induced by agar culture, and to the liberation from agar of colonies and clusters at the time of harvest. The reader should recognize that our calculation of the enhancement of mitoses due to this procedure can, at best, be only an estimate, because of the difficulty of calculating recovery of morphological entities from agar and the extreme scarcity of mitoses in direct preparations. However, by using an image analyser we have calculated mitotic index on the basis of the total number of cells per sample. These results are presented in Table II. Examination of intact plating layers has demon-

TABLE II.—*Mitotic index* of human tumour cells by direct, liquid, and agar culture techniques*

| Sample | Tumour type | Direct | Liquid | Agar |
|---|---|---|---|---|
| 1 | Adenocarcinoma breast | 0·0022 | 0·0010 | 0·17 |
| 2 | Adenocarcinoma ovary | 0·0070 | — | 1·70 |
| 3 | Adenocarcinoma ovary | — | 0·0030 | 0·60 |
| 4 | Adenocarcinoma ovary | 0·0032 | — | 0·28 |
| 5 | Adenocarcinoma breast | 0·0022 | 0·0041 | 0·04 |

* $\frac{\text{Total of mitotic figures}}{\text{Total cells}} \times 100$

strated that mitoses are almost exclusively localized to generating colonies and clusters. However, disaggregation of a small number of mitoses from colonies and clusters can occur during the processing steps. If the enhancement in tumour mitoses in agar culture is calculated on the basis only of cells in clusters or colonies (the clonogenic and therefore proliferative fraction) a mitotic index of up to 9% has been observed. Thus, the increase in the mitotic index beyond direct preparations is further multiplied, up to several thousand-fold. Although direct tumour samples from some malignant ascites may contain a large proliferative compartment

FIG. 2.—Banded chromosomes from tumour cells of Patient EV. (A) G-banded metaphase displaying a variety of complex chromosome changes (see Fig. 3); (B) C-banded metaphase displaying heavily stained regions of constitutive heterochromatin; (C) N-banding showing silver staining of nucleolus-organizer regions. Metacentric as well as the normal acrocentric silver staining is observed (arrows). (D) Multiple copies of double minute bodies (arrows) in addition to numerous tumour chromosomes, stained by Giemsa.

FIG 3.—G-banded karotype from Patient EV. A variety of chromosome abnormalities, including: del(1)(pter→q25:), del(2)(pter→q23:), del(6)(pter→q15:), +4 unidentified markers.

(*e.g.* MI = 1%) this occurrence is extremely rare (Kakati & Sandberg, 1978). The numerical enhancement observed from our studies indicates substantially increased tumour cell recovery in a large number and wide variety of solid and ascitic tumours. Additionally, in the 5 cases in which direct harvesting and colony analysis have been compared, similarities in modal number, structural variants, and marker chromosomes have been found.

Examples of the application of G-, C-, and N-banding to tumour cells grown 72 h in the colony-assay system are depicted in Fig. 2. Tumour cells were procured from patient E.V., a 47-year-old woman in whom the diagnosis of ovarian adenocarcinoma had just been established and had not received treatment with chemotherapy or radiotherapy. Samples from the patient's primary solid tumour and a malignant ascites were obtained simultaneously, and cultured separately with the stem-cell assay. Giemsa analysis revealed a modal count of 38 in both direct harvest and colony samples, with G-banding revealing a wealth of karyotypic variability. The most common karyotypic aberrations within the multiple stem lines present in this tumour were: 38, -X, -17, -22, del(1)(pter→q25:), del(2)(pter→q23:), del(6)(pter→q15:), +1–4 unidentified marker chromosomes (Fig. 3). The observation in this tumour of at least 4 major karyotypically unique progenitor-cell populations is consistent with the suggestion that our assay system captures a representative sample of the clonogenic fraction within tumour samples. The structural and numerical intra-tumour chromosome variation between clonogenic populations suggests that substantial karyotypic "evolution" (progressive clonal heterogeneity) (Nowell, 1977) has already occurred in the stem-cell pool of the tumour from this untreated patient. The ability to perform chromosome-banding analysis by G-banding (Fig. 2a) to demonstrate constitutive heterochromatin by C-banding (Fig. 2b) and selectively to silver-stain transcriptionally active cistrons for 18S and 28S ribosomal RNA by N-banding (Fig. 2c) was extremely useful in characterizing the complexly rearranged tumour-cell chromosomes in this cancer. Chromosome-banding of direct samples from this patient, although similar in modal number, provided far less information, owing to an extremely low mitotic index and morphologically sub-optimal chromosomes. Interestingly, cytogenetic comparisons between the colony-forming tumour cells in both the patient's ascites and solid-tumour biopsy sample revealed a similar modal chromosome number, though a higher percentage of polyploidy was found in tumour cells from the primary site.

In addition to the marked structural chromosomal variation between this patient's tumour cells, ~1% of all mitoses displayed dozens of double minute bodies (dms) (Fig. 2d). The finding of dms in tumour cells from cluster and colony samples from this patient is strong support for the neoplastic origin of these cells (Barker & Hsu, 1979). The occurrence of dms and acquired drug resistance have recently been associated with specific gene amplification in established animal tumour lines (Alt *et al.*, 1978). Cytogenetic analysis of spontaneous human tumours may display similar or additional changes in relation to resistance to methotrexate or other anticancer drugs. Cytogenetic comparisons between the clonogenic fractions before and after chemotherapy in our system may facilitate identification of unique progenitor cells resistant to specific anticancer drugs.

The procurement of chromosomes from human solid tumours by direct harvesting techniques has normally failed to provide mitotic figures morphologically suitable for Giemsa-banding. The effects of this restriction on human solid-tumour karyology is profound. Although epithelial cancers are the most common of human tumours, they have rarely been analysed with chromosome-binding techniques. Recent reviews have shown that less than 5% of all banded chromosomal analyses of human tumours published to date have involved carcinomas (Mitelman & Levan, 1978). With increasing clinical application of cytogenetics to human malignancies (Golomb *et al.*, 1978; Trent & Davis, 1979) detailed studies using chromosome-banding of human epithelial cancers is needed. Methodological improvement in the cytogenetic analysis of solid tumours and their ascites has long been sought. Use of our method should greatly facilitate detailed karyotypic assessment of a variety of human tumours and may provide important new basic and clinical observations relevant to cancer.

The authors gratefully acknowledge the scientific input and provision of cultures by Anne Hamburger, Ph.D., Ronald Buick, Ph.D., Barbara Soehnlen, John R. Davis, M.D., Debbie Saxe, and technical support of Yvette Frutiger, Laurie Young, Steve Fry and Iris Veomett. Daniel Von Hoff, M.D., Department of Medicine, The University of Texas at San Antonio, kindly provided biomarker data from his neuroblastoma studies (D. Von Hoff *et al.* (1979) *Am. Assoc. Cancer Res.*, **20**, 51). Clinical samples were kindly provided by David Alberts, M.D., Brian Durie, M.D., Terence Herman, M.D., Stephen Jones, M.D., Leo McMahon, M.D., Frank Meyskens, M.D., and Thomas Stanisic, M.D. Technical assistance in image analysis was provided by Mr Bernie Kressner, Bausch and Lomb, Rochester, New York.

The authors' research is supported in part by Grants CA-21839, CA-17904, and CA-23074 from the National Cancer Institute, National Institutes of Health, Bethesda, Maryland 20205.

### REFERENCES

Alt, F., Kellens, R., Bertino, J. & Schimke, R. (1978) Selective multiplication of dihydrofolate reductase genes in methotrexate-resistant variants of cultured murine cells. *J. Biol. Chem.*, **253**, 1357.

BARKER, P. & HSU, T. C. (1979) Double minutes with special references to breast carcinomas. *J. Natl Cancer Inst.*, **62**, 257.

GOLOMB, H., VARDINAR, J., ROWLEY, J., TESTA, J. & MINTZ, J. (1978) Correlation of clinical findings with quinacrine-banded chromosomes in 90 adults with acute non-lymphocytic leukemia. *N. Engl. J. Med.*, **299**, 613.

GOODPASTURE, C. & BLOOM, S. (1975) Visualization of nucleolar organizer regions in mammalian chromosomes using silver staining. *Chromosoma*, **53**, 37.

HAMBURGER, A. W. & SALMON, S. E. (1977*a*) Primary bioassay of human tumor stem cells. *Science*, **197**, 461.

HAMBURGER, A. W. & SALMON, S. E. (1977*b*) Primary bioassay of human myeloma stem cells. *J. Clin. Invest.*, **60**, 846.

HAMBURGER, A. W., SALMON, S. E., KIM, M. B. & 4 others (1978) Direct cloning of human ovarian carcinoma cells in agar. *Cancer Res.*, **38**, 3438.

JONES, S. E., HAMBURGER, A. W., KIM, M. B. & SALMON, S. E. (1979) Development of a bioassay for putative human lymphoma stem cells. *Blood*, **53**, 294.

KAKATI, S., HAYATA, I., OSHIMINO, M. & SANDBERG, A. (1975) Chromosomes and causations of human cancer and leukemia: X banding patterns in cancerous effusions. *Cancer*, **36**, 1729.

KAKATI, S. & SANDBERG, A. (1978) Chromosomes in solid tumors. *Virchows. Archiv* B. [Cell. Pathol.], **29**, 129.

METCALF, D. (1977) *Hemopoietic colonies.* Berlin: Springer Verlag.

MILLER, D. A., TANTRAVAKI, R., DEN, V. & MILLER, O. G. (1976) Q- and C-band chromosome markers in inbred strains of *Mus musculus*. *Genetics*, **84**, 67.

MITELMAN, F. & LEVAN, G. (1978) Clustering of aberrations to specific chromosomes in human neoplasms. III Incidence and geographic distribution of chromosome aberrations in 856 cases. *Hereditas*, **89**, 207.

MOORE, M. A. S. & METCALF, D. (1973) Cytogenetic analysis of human acute and chronic myeloid leukemia cells cloned in agar culture. *Int. J. Cancer*, **11**, 143.

NAJFELD, V., SINGER, M., JAMES, M. & FIALKOW, P. (1978) Trisomy 1q in preleukemia with progression to acute leukemia. *Scand. J. Haematol.*, **21**, 24.

NOWELL, P. (1977) Preleukemia: Cytogenetic clues in some confusing disorders. *Am. J. Pathol.*, **89**, 459.

SALMON, S. E. & BUICK, R. N. (1979) Preparation of permanent slides of intact soft agar colony cultures of hematopoietic and tumor stem cells. *Cancer Res.*, **39**, 1133.

SALMON, S. E. & LIU, R. (1979) Direct "wet" staining of tumour or haematopoietic colonies in agar culture. *Br. J. Cancer*, **39**, 779.

SHILOH, Y. & COHEN, M. (1978) An improved technique of preparing bone-marrow specimens for cytogenetic analysis. *In Vitro*, **14**, 510.

SUN, N., CHU, E. & CHANG, C. (1973) Staining method for the banding patterns of human mitotic chromosomes. *Mamm. Chromosome Newsl.*, **14**, 26.

TRENT, J. M. & DAVIS, J. R. (1979) D-group chromosome abnormalities in endometrial cancer and hyperplasia. *Lancet*, ii, 361.